# Data regarding 2D:4D and other digit ratios in Greek population

**DOI:** 10.1016/j.dib.2021.106724

**Published:** 2021-01-09

**Authors:** Ioannis Kyriakidis

**Affiliations:** Hematology Oncology Unit, 2nd Pediatric Department, Aristotle University of Thessaloniki, University General Hospital AHEPA, Thessaloniki, Greece

**Keywords:** Digit ratio, 2d:4d, Sexual dimorphism, Prenatal exposure, Sex hormones, Environmental estrogens, DDT

## Abstract

Second–to–fourth digit ratio (2D:4D) is a sexually dimorphic biometric marker, which is influenced by prenatal estrogen levels and reflects to the hormonal profile of each individual. Exposure to environmental estrogens was common in the past. Their endocrine–disrupting action, combined with their long half–time, may have a feminizing effect and an impact on health. A stratified sample of 160 Greek people was selected by random procedures and fingers’ length was measured by means of an electronic caliper. Based on preliminary statistical analysis, middle aged persons, corresponding to birth in the period 1947–1972, were found to differ significantly from the rest and the original sample was subsequently divided into three age subgroups (≤37, 38–63 and ≥64 years old). 2D:4D ratio was found significantly higher in Greek people aged from 38 to 63 years old, although sexual dimorphism remained unaffected. The other digit ratios followed the same pattern, with people aged less than 38 years to share equivalent ratios with people aged more than 63 years.

## Specifications Table

**Table:** 

Subject	Health and medical sciences
Specific subject area	Digit ratios in Greek population
Type of data	Tables: 2 Table 1. Mean digit ratio measurements and significant differences between age subgroups for female subjects. Table 2. Mean digit ratio measurements and significant differences between age subgroups for male subjects. Datasets in Medeley Data: https://data.mendeley.com/drafts/fbdbh7g29v
How data were acquired	The research was performed in accordance with the principles of the Declaration of Helsinki. All participating persons gave their consent to the study, which was approved by the Faculty Ethics Committee. A vernier caliper with precision of 0.01 mm was used by two independent researchers. Biostatistical analysis was performed by means of the statistical package SPSS 17.0 (SPSS Inc, Chicago, IL).
Data format	Raw in Medeley Data. Analyzed in text.
Parameters for data collection	The participants and the persons taking measurements were ignorant of the physiological meaning of 2d:4d ratio. Individuals with former hand lesions, endocrinopathies, chromosomal abnormalities and those who identified themselves as homosexual or bisexual or left–handed were excluded.
Description of data collection	All fingers of both hands, except from the thumbs, were measured directly by means of an electronic vernier caliper (accuracy 0.01 mm; DV 892, LCD digital caliper, Tecnotest S.R.L., Modena, Italy). The length of each finger was defined as the distance from the middle of the ventral proximal crease of the digit to its tip. If there was a band of creases, measurements were made from the most proximal of these. Measurements were performed twice, with the second measurement made blinded to the first. Finger lengths were calculated as the mean values of the first and second measurement of the length of each digit.
Data source location	Institution: Aristotle University of Thessaloniki City/Town/Region: Thessaloniki Country: Greece Latitude and longitude (and GPS coordinates, if possible) for collected samples/data: Latitude 40° 37′ 28.79" N, longitude: 22° 57′ 17.39" E
Data accessibility	Row data are hosted in the trusted repository. Repository name: Mendeley Data Data identification number: http://dx.doi.org/10.17632/fbdbh7g29v.1 Direct URL to data: https://data.mendeley.com/drafts/fbdbh7g29v
Related research article	For a co-submission (when your related research article has not yet published): I. Kyriakidis. Exposure to environmental estrogens and second–to–fourth digit ratio in Greek population: A possible link. Early Human Development https://doi.org/10.1016/j.earlhumdev.2020.105245

## Value of the Data

• Second-to-fourth digit ratio (2D:4D) is a useful biometric marker and has been correlated with a variety of diseases and conditions. 2D:4D ratios vary among ethnic groups and datasets from around the world can help future studies. Measurements of other digit ratios are lacking in published literature but they might have potential clinical interest so they are hereby provided.
• Researchers with special interest on digit ratios will find useful to compare data of digit ratios other than 2D:4D. Data from digit ratios among specific age groups would be also of benefit for future research.
• Checking for outliers in digit ratios’ data can reveal outliers that should be properly explained. Study of other digit ratios can reveal associations that are not significant with 2D:4D ratio.

## 1 Data Description

Raw data have been deposited in Mendeley Data: http://dx.doi.org/10.17632/fbdbh7g29v.1. Raw data: (A) Sex: 0 = Female, 1 = Male; (B) D = digit length in mm e.g. 2D is the second digit; (C) Side: R = right hand; L = left hand e.g. L2D is left second digit; (D) MI = myocardial infarction; (E) when both measurements were typed in the file ***_1 and ***_2 reflect the first and the second measurement respectively; (F) Age is measured in years; (G) Height is in meters; (H) BMI = body mass index; (I) WHR = waist-to-hip ratio; (J) Weight in Kg; (K) Waist and circumference in cm.

*Primary analysis*. No significant difference was found between each individual's first and second measurement of digits’ length. Inter–individual differences were significantly greater (p<0.01) than measurement errors in all digit ratios (i.e. the differences between two successive measurements of each ratio), so the calculated values of digit ratios do reflect real differences among individuals. Women had all their fingers significantly shorter than men (p<0.001), a fact that is attributed to the respective difference in median heights. Boxplots for all digit lengths and calculated digit ratios presented no outliers and the values’ distributions were normal (Kolmogorov–Smirnov normality test, p>0.05). Females were found with significantly higher (p<0.001) 2d:3d, 2d:4d and 2d:5d ratios for both hands than males. Comparatively, less significant differences were calculated as well for 4d:5d ratio of the right hand and 3d:4d ratio of the left hand (with females having higher ratios; p=0.04 and p=0.015 respectively). Pearson correlation coefficient for 2d:4d and age presented no significant correlation. On the contrary, the scatterplot of 2d:4d ratio and age revealed that 2d:4d might be dependent on age. Specifically, an increase of 2d:4d ratio was observed for middle aged Greeks. Combining linear least squares regression and Loess modelling method (Epanechnikov kernel; 50% of points to fit) [1], we deduced that the increase of 2d:4d ratios referred mainly to subjects aged from 38 to 63 years.

Subsequently, the initial sample was divided in three age groups. The first age group consisted of 40 subjects, aged from 19 to 37 years (20 females and 20 males; mean age: 27.7 ± 4.5 for females and 27.5 ± 3.5 for males). The most significant difference was measured for 2d:4d ratio of the right hand, with women having higher ratio (p<0.001). Sexual dimorphism (p<0.05) was also present for 2d:3d and 2d:5d ratios of the right hand, as well as for 2d:3d, 2d:4d and 3d:5d ratios of the left hand. Age and 2d:4d ratio presented no significant correlation (rP=-0.026; p=0.924). The current analysis derived from combination of datasets belonging to earlier studies [2], [3], [4], [5], [6].

The second age group consisted of 60 subjects, aged from 38 to 63 years (30 females and 30 males; mean age: 50.7 ± 7.5 for females and 51.1 ± 7.7 for males). Sexual dimorphism in this group was more prominent, with seven out of twelve ratios to differ significantly. Both hands’ 2d:3d and 2d:4d ratios were significantly higher in females (p<0.001). Moreover, significant differences were measured for 2d:5d (p<0.001 for the right and p=0.002 for the left hand) and right hand's 4d:5d ratio (p=0.028). Age and 2d:4d ratio did not correlate each other (rP=-0.088; p=0.502).

The third group consisted of 60 subjects, aged from 64 to 91 years (30 females and 30 males; mean age: 75.6 ± 6.9 for females and 75.6 ± 8.5 for males). Significant differences between sexes were found for 2d:3d (p<0.001 for the right and p=0.006 for the left hand), 2d:4d (p<0.001 for the right and p=0.01 for the left hand) and 2d:5d ratios (p<0.001 for the right hand). Correlation between age and 2d:4d presented no statistical significance (rP=-0.016; p=0.906).

The investigation on the presence of sexual dimorphism in each age group was succeeded by comparisons of mean digit ratios between the three age groups. Tables 1 and 2, present these differences. Notably, middle aged Greeks had significantly higher 2d:4d and 2d:3d ratios in both hands than young and aged Greeks. In particular, women of the second age group had higher 2d:4d ratios (1.036 ± 0.048 for the right hand and 1.043 ± 0.041 for the left hand) than women of the first age group (1.003 ± 0.024 for the right hand and 1.008 ± 0.035 for the left hand; p=0.003 and p=0.002 respectively) or the third one (1.014 ± 0.032 for the right and 1.013 ± 0.062 for the left hand; p=0.047 and p=0.027 respectively). As regards middle aged men, they also had higher 2d:4d ratios (0.993 ± 0.041 for the right and 0.997 ± 0.027 for the left hand) than men of the first age group (0.97 ± 0.027 for the right and 0.979 ± 0.029 for the left hand; p=0.034 and p=0.025 respectively) or the third one (0.972 ± 0.032 for the right and 0.98 ± 0.021 for the left hand; p=0.031 and p=0.009 respectively). Neither in men, nor in women, was detected significant difference between 2d:4d ratios of the first and the third age group.

**Table 1:** Mean digit ratio measurements and significant differences between age subgroups for female subjects.

	1^st^ age group		2^nd^ age group		3^rd^ age group	
	aged ≤ 37 (n = 20)	1^st^ and 2^nd^ group	aged 38 – 63 (n = 30)	2^nd^ and 3^rd^ group	aged ≥ 64 (n = 30)	1^st^ and 3^rd^ group
Digit ratios	Mean (±SD)	difference (p)	Mean (±SD)	difference (p)	Mean (±SD)	difference (p)
R 2d:3d	0.922 (±0.021)	<0.001	0.976 (±0.033)	0.002	0.949 (±0.032)	0.001
R 2d:4d	1.003 (±0.024)	0.003	1.036 (±0.048)	0.047	1.014 (±0.032)	NS
R 2d:5d	1.224 (±0.05)	0.007	1.27 (±0.06)	NS	1.248 (±0.052)	NS
R 3d:4d	1.087 (±0.024)	0.022	1.061 (±0.051)	NS	1.069 (±0.035)	NS
R 3d:5d	1.327 (±0.062)	NS	1.301 (±0.058)	NS	1.316 (±0.061)	NS
R 4d:5d	1.221 (±0.047)	NS	1.227 (±0.058)	NS	1.231 (±0.046)	NS
L 2d:3d	0.919 (±0.023)	<0.001	0.97 (±0.03)	NS	0.955 (±0.045)	0.001
L 2d:4d	1.008 (±0.035)	0.002	1.043 (±0.041)	0.027	1.013 (±0.062)	NS
L 2d:5d	1.216 (±0.053)	0.044	1.248 (±0.054)	NS	1.228 (±0.079)	NS
L 3d:4d	1.097 (±0.04)	NS	1.076 (±0.041)	NS	1.061 (±0.058)	0.013
L 3d:5d	1.323 (±0.05)	0.018	1.287 (±0.052)	NS	1.286 (±0.08)	0.048
L 4d: 5d	1.207 (±0.045)	NS	1.197 (±0.045)	NS	1.214 (±0.072)	NS

R = right hand, L = left hand, SD = standard deviation and NS = not significant difference (p>0.05).

**Table 2:** Mean digit ratio measurements and significant differences between age subgroups for male subjects.

	1^st^ age group		2^nd^ age group		3^rd^ age group	
	aged ≤ 37 (n = 20)	1^st^ and 2^nd^ group	aged 38 – 63 (n = 30)	2^nd^ and 3^rd^ group	aged ≥ 64 (n = 30)	1^st^ and 3^rd^ group
Digit ratios	Mean (±SD)	difference (p)	Mean (±SD)	difference (p)	Mean (±SD)	difference (p)
R 2d:3d	0.908 (±0.023)	0.05	0.924 (±0.032)	0.055	0.91 (±0.025)	NS
R 2d:4d	0.97 (±0.027)	0.034	0.993 (±0.041)	0.031	0.972 (±0.032)	NS
R 2d:5d	1.186 (±0.041)	NS	1.186 (±0.064)	NS	1.185 (±0.04)	NS
R 3d:4d	1.069 (±0.032)	NS	1.074 (±0.027)	NS	1.069 (±0.03)	NS
R 3d:5d	1.307 (±0.046)	NS	1.284 (±0.064)	NS	1.303 (±0.041)	NS
R 4d:5d	1.223 (±0.039)	0.05	1.195 (±0.053)	0.038	1.22 (±0.037)	NS
L 2d:3d	0.949 (±0.027)	0.011	0.926 (±0.032)	NS	0.928 (±0.027)	0.09
L 2d:4d	0.979 (±0.029)	0.025	0.997 (±0.027)	0.009	0.98 (±0.021)	NS
L 2d:5d	1.195 (±0.053)	NS	1.197 (±0.068)	NS	1.197 (±0.054)	NS
L 3d:4d	1.072 (±0.035)	NS	1.078 (±0.034)	NS	1.077 (±0.031)	NS
L 3d:5d	1.261 (±0.058)	NS	1.293 (±0.066)	NS	1.29 (±0.048)	NS
L 4d:5d	1.222 (±0.041)	NS	1.201 (±0.063)	NS	1.221 (±0.047)	NS

R = right hand, L = left hand, SD = standard deviation and NS = not significant difference (p>0.05).

A two–way between–groups analysis of variance was conducted to explore the impact of sex and age on the right hand's 2d:4d ratio. There was a statistically significant main effect for age [F(2,154)=8.58, p<0.001] and the effect size was moderate to large (eta squared=0.1). Post–hoc comparisons using the Tukey Honestly Significant Difference test indicated that the mean score for the 19–37 years age group (M=0.986, SD=0.03) did differ significantly (p=0.001) from the 38–63 years age group (M=1.014, SD=0.049). The first age group differed also significantly (p=0.004) from the 64–91 years age group (M=0.993, SD=0.038). No significant difference was estimated between the first and the third age group. The main effect for sex [F(1,154)=46.03, p<0.001] differed significantly and the effect size was large (eta squared=0.23), as expected, while the interaction effect [F(2,154)=0.26, p=0.768] did not reach statistical significance.

Age, sex and 2d:4d ratios were subjected to factor analysis and the results were in favour of the theory that both age and sex play crucial roles in the differences observed in digit ratios.

The findings of the current paper are, in general, in agreement with former studies made on the field [7]. Greek subjects have been included in similar studies before [2,3], while data reported here correspond to a recent publication [8]. Women were found with significantly higher 2d:4d ratios than men (1.019±0.039 versus 0.979±0.036 for the right hand and 1.023±0.051 versus 0.986±0.026 for the left hand; p<0.001). As expected, sexual dimorphism remained unaffected, even when our initial sample was divided into three age subgroups (Tables 1 and 2).

The innovation of this dataset is that subjects were measured from a wide range of ages (from 19 to 91 years old). Primary analysis showed that 2d:4d ratio and age might correlate each other. On the contrary, in all three age subgroups Pearson correlation coefficient for 2D:4D ratio of the right hand and age ranged from -0.088 to -0.016. The latter means that 2D:4D ratios remain stable in between each group and, given that sexual dimorphism was evident in all groups, the differences observed between the mean ratios of the three groups point out the presence of some factor that affected upwards the digit ratios of both sexes in the second age group. The increase of 2d:4d ratio in the second group was greater for females (2.6 % for the right and 3.2 % for the left hand) than for males (2.2 % for the right and 1.8 % for the left hand), suggesting that the involved environmental factor was rather estrogenic (or anti–androgenic) than androgenic. Additionally, middle aged men presented so “feminized” –or “demasculinized”– 2d:4d ratios that they bore no significant difference with those of young women, even though they differed significantly (p<0.001) from middle aged women. This discontinuity led us to the endocrine–disrupting theory, as the most plausible explanation.

## 2 Experimental Design, Materials and Methods

Subjects. A stratified sample of 160 Greek adults (80 females and 80 males, age range 19 – 91 years, mean age 54.3 for females and 54.4 for males) was selected by random procedures. The participants and the persons taking measurements were ignorant of the physiological meaning of 2D:4D ratio. Individuals with former hand lesions, endocrinopathies, chromosomal abnormalities and those who identified themselves as homosexual or bisexual or left–handed were excluded [9].

Measurements of digits’ length. All fingers of both hands, except from the thumbs, were measured directly by means of an electronic vernier caliper (precision 0.01 mm). The length of each finger was defined as the distance from the middle of the ventral proximal crease of the digit to its tip. If there was a band of creases, measurements were made from the most proximal of these. Measurements were performed twice, with the second measurement made blinded to the first. Finger lengths were calculated as the mean values of the first and second measurement of the length of each digit.

Biostatistical analysis was performed by means of the statistical package SPSS 17.0 (SPSS Inc, Chicago, IL).

## Ethics Statement

The research was performed in accordance with the principles of the Declaration of Helsinki. All participating persons gave their consent, which was approved by the Faculty Ethics Committee.

## Declaration of Competing Interest

None.
